# Simplified Double-Pulley Remplissage With Knotless All-Suture Anchor Fixation Using a Digital Subdeltoid Retrieval Method

**DOI:** 10.1016/j.eats.2025.103631

**Published:** 2025-05-23

**Authors:** Cody Ashy, J. Ambrose Martino, Mitchell H. Negus, Brandon L. Rogalski, Richard J. Friedman, Josef K. Eichinger

**Affiliations:** Department of Orthopaedics and Physical Medicine, Medical University of South Carolina, Charleston, South Carolina, U.S.A.

## Abstract

Anterior shoulder dislocations represent one of the most common joint dislocations, with particular populations at increased risk of recurrence. Associated bone loss further increases the risk of dislocation and often needs to be addressed via soft tissue or bony augmentation. In particular, for large Hill-Sachs lesions in patients at increased risk of dislocation, remplissage is indicated. This current technique builds upon previously double-pulley techniques and utilizes a knotless all-suture anchor construct that allows for maximization of anchor spread within an associated Hill-Sachs lesion. The knotless all-suture anchor constructs are placed percutaneously under direct visualization through a routine posterior portal. After completion of any associated labral repair, the posterior incision is expanded, and the sutures are retrieved via digital dissection to facilitate a double-pulley repair. The benefits of this technique include maximization of anchor spread, use of an already established routine portal, forgoing time-consuming arthroscopic access to the subacromial space, and a knotless construct that optimizes outcomes without compromising strength. Additionally, retrieval of the sutures under direct visualization through the expanded posterior portal decreases the likelihood of tissue bridge formation.

Anterior shoulder dislocations remain one of the most common joint dislocations in the body,^1^, ^2^, ^3^ and in particular, young male athletes participating in contact or overhead throwing sports are at risk, with rates as high as 3% per year.^4^^,^^5^ Given the risk of recurrence, particularly in at-risk populations, there has been an increased emphasis on the surgical management of these injuries, even in first-time dislocations.^6^, ^7^, ^8^ Associated with anterior shoulder instability is posterior humeral head bone loss, known as a Hill-Sachs lesion. These lesions occur in up to 40% to 90% of shoulder dislocations.^9^, ^10^, ^11^ The size of Hill-Sachs lesions and associated glenoid bone loss determine if they are “on-track” or “off-track” lesions, with “off-track” lesions resulting in increased dislocation risk as the Hill-Sachs lesion can lever the humerus out of the socket.^12^^,^^13^ Consequently, Hill-Sachs lesions must be considered when discussing open versus arthroscopic shoulder stabilization and considering soft tissue versus bony augmentation of stabilization procedures.

A remplissage procedure is an extracapsular tenodesis that fixates the infraspinatus tendon and shoulder capsule into the Hill-Sachs lesion to prevent leverage of the humeral head out of the socket.^11^ Classically, remplissage procedures were indicated for patients with subcritical bone loss and an off-track lesion.^14^ However, recent literature has highlighted that the addition of a remplissage improves return-to-sport rates compared with isolated Bankart repairs alone.^15^, ^16^, ^17^, ^18^

An arthroscopic double-pulley remplissage technique was originally described by Woodall and has since been elaborated on by several authors.^19^, ^20^, ^21^, ^22^ However, these techniques vary in the anchors used, both all-suture and hard-bodied anchors, as well as the method of anchor placement. This current technique builds upon previous double-pulley techniques and utilizes a knotless all-suture anchor construct, which allows for maximization of anchor spread within the Hill-Sachs lesion via percutaneous placement of anchors and shuttling sutures through a single, previously established, portal.

## Surgical Technique

### Preoperative Workup

Obtain standard radiographs preoperatively, including Grashey, scapular Y, and axillary lateral. In patients with recurrent dislocations or symptomatic instability following conservative management, obtain a magnetic resonance arthrogram to assess for labral pathology. For those with suspected or identified bony defects on radiographic assessments, preoperative computed tomography can be considered to better assess whether the lesion is “on-track” or “off-track.”

### Intraoperative Procedure

First, the patient is placed in the lateral decubitus position on a peg board, with a slight posterior tilt. In the lateral decubitus position, an examination under anesthesia is performed with a load-and-shift test to assess for the degree of instability. Routine sterile draping is performed, and the arm is secured using the SPIDER2 Limb Positioner (Smith & Nephew). The arm is positioned so that it is pulled in line down the body in approximately 35° of abduction with a slight distraction force to facilitate entry into the joint.

**Fig 1:**
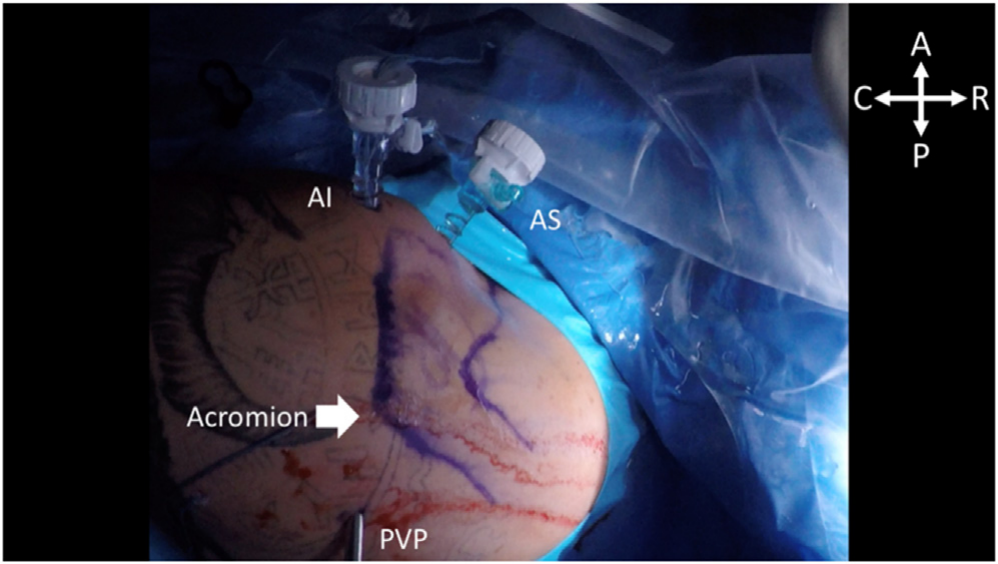
With the patient positioned in the right lateral decubitus position, the acromion is marked out with blue ink, as indicated by the white arrow. The patient’s head is toward the right-hand side of the figure while the patient’s arm is suspended toward the left-hand side of the figure. The posterior viewing portal (PVP) is inferomedial to the posterolateral edge of the acromion. The 7-mm Arthrex Twist-in Cannula and 5.5-mm Arthrex Instrument Cannula can be seen in the anteroinferior (AI) and anterosuperior (AS) portals, respectively. (A, anterior; C, caudal; P, posterior; R, rostral.)

Preoperative landmarks are drawn, including the outline of the acromion, scapular spine, clavicle, and coracoid. A standard posterior portal is made approximately 2 finger breadths inferior and 2 finger breadths medial to the inferolateral edge of the acromion. A diagnostic arthroscopy is performed, and standard anterosuperior and anteroinferior portals are made in accordance with surgeon preference to address the Bankart lesion. In this case, a 7-mm Arthrex Twist-in Cannula and a 5.5-mm Arthrex Instrument Cannula are placed within the anteroinferior and anterosuperior portals, respectively. Figure 1 shows the positioning of the patient and initial portal placement.

**Fig 2:**
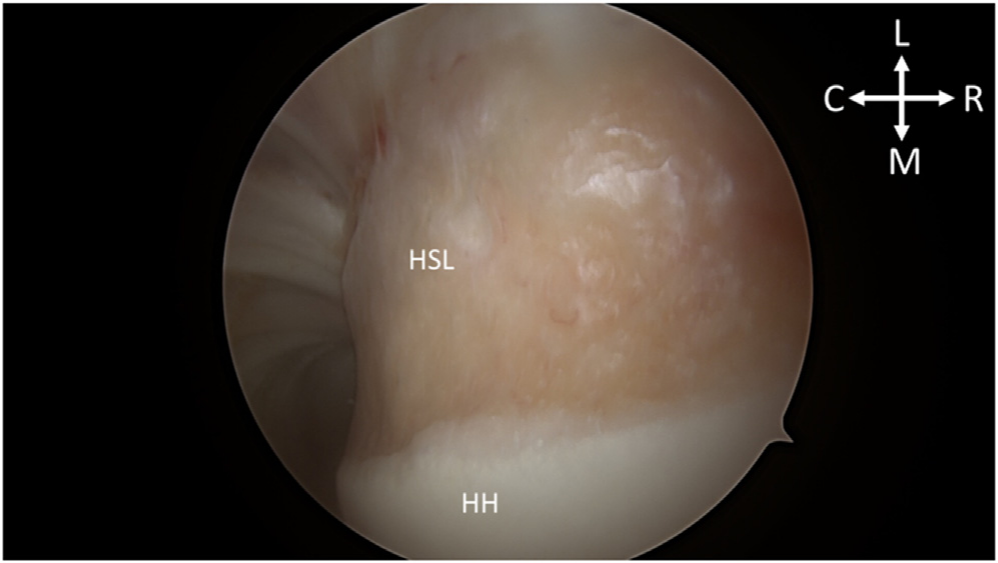
With the patient in the right lateral decubitus position and while viewing from the posterior portal, the Hill-Sachs lesion is identified from the posterior viewing portal, just adjacent to the humeral head cartilage surface. (C, caudal; HH, humeral head; HSL, Hill-Sachs lesion; L, lateral; M, medial; R, rostral.)

**Fig 3:**
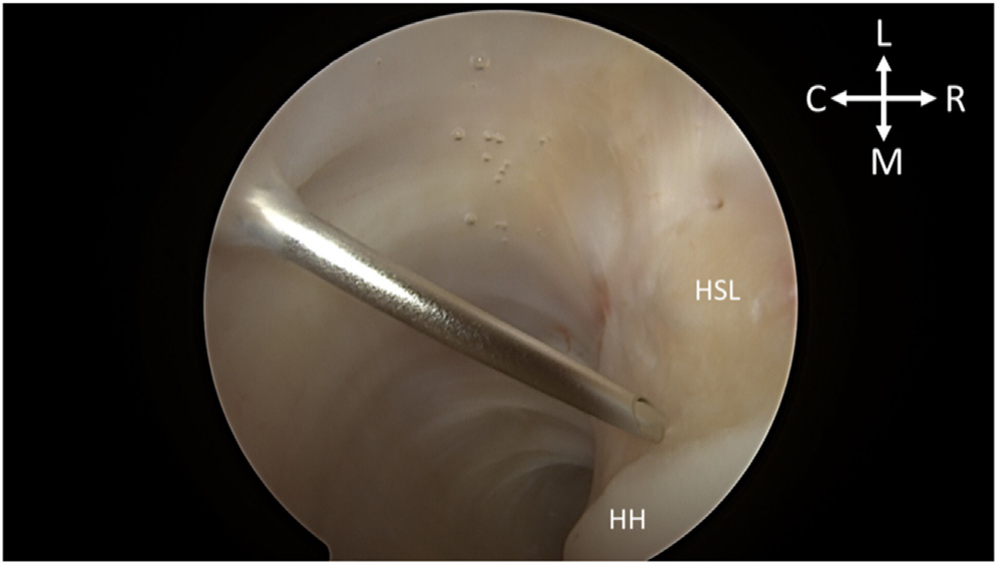
With the patient in the right lateral decubitus position and while viewing from the posterior portal, the first of the 2 remplissage anchors is planned for insertion via spinal needle localization. (C, caudal; HH, humeral head; HSL, Hill-Sachs lesion; L, lateral; M, medial; R, rostral.)

**Fig 4:**
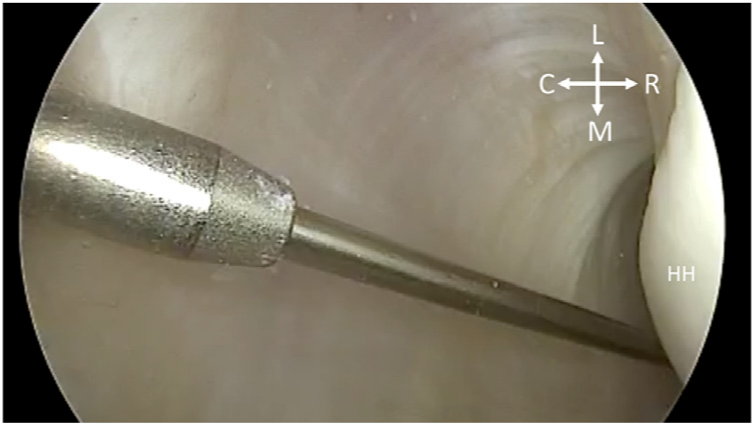
With the patient in the right lateral decubitus position and while viewing from the posterior portal, the soft tissue dilator is placed over the nitinol wire to facilitate safe entry of the drill guide for anchor placement, taking care to aim away from the humeral head cartilage into the axillary pouch. (C, caudal; HH, humeral head; L, lateral; M, medial; R, rostral.)

**Fig 5:**
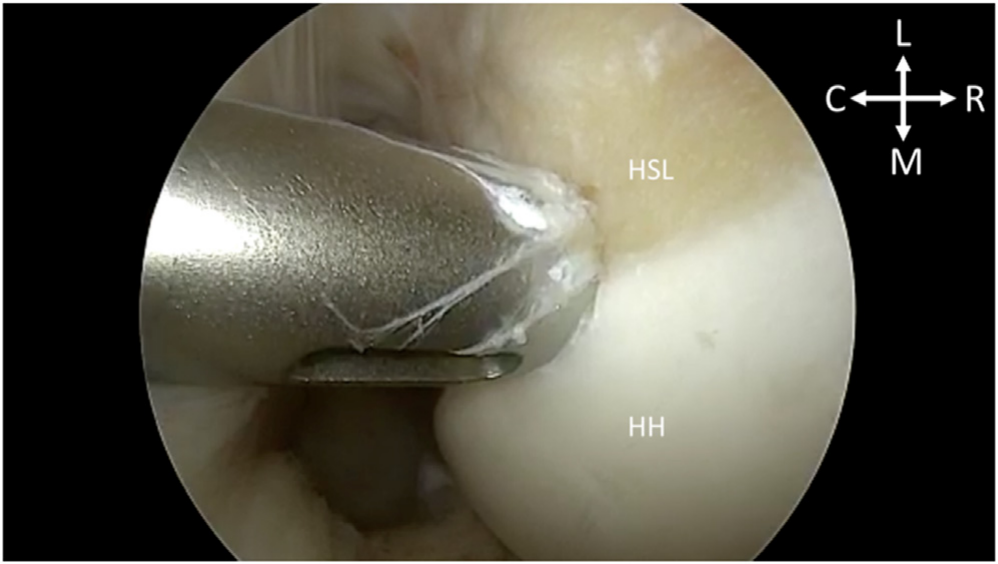
With the patient in the right lateral decubitus position and while viewing from the posterior portal, the drill guide for the Knotless 2.6-mm FiberTak (Arthrex) is placed in the caudal aspect of the Hill-Sachs lesion just adjacent to the humeral head cartilage. (C, caudal; HH, humeral head; HSL, Hill-Sachs lesion; L, lateral; M, medial; R, rostral.)

**Fig 6:**
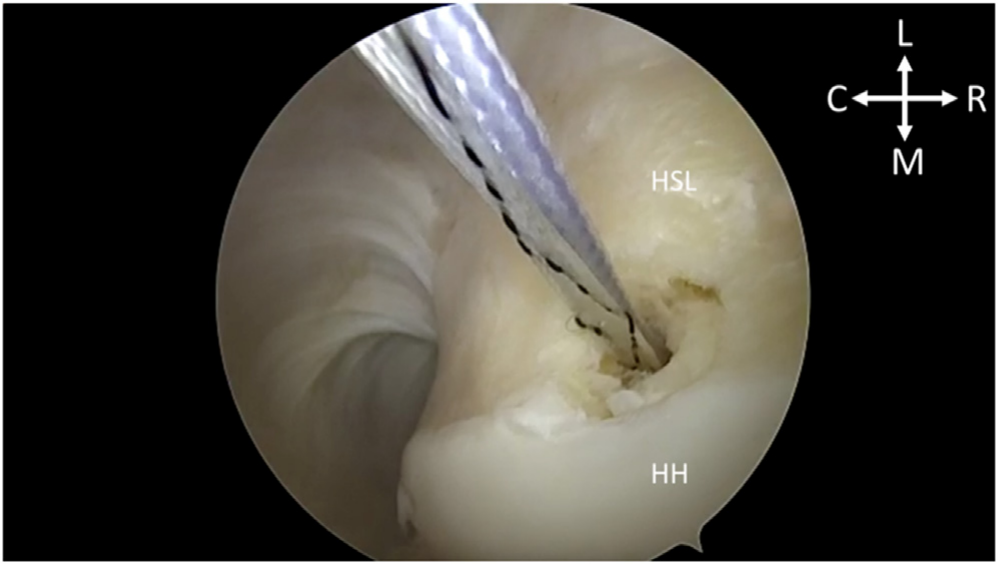
With the patient in the right lateral decubitus position and while viewing from the posterior portal, the caudal anchor is placed and successfully tensioned within the Hill-Sachs lesion. (C, caudal; HH, humeral head; HSL, Hill-Sachs lesion; L, lateral; M, medial; R, rostral.)

**Fig 7:**
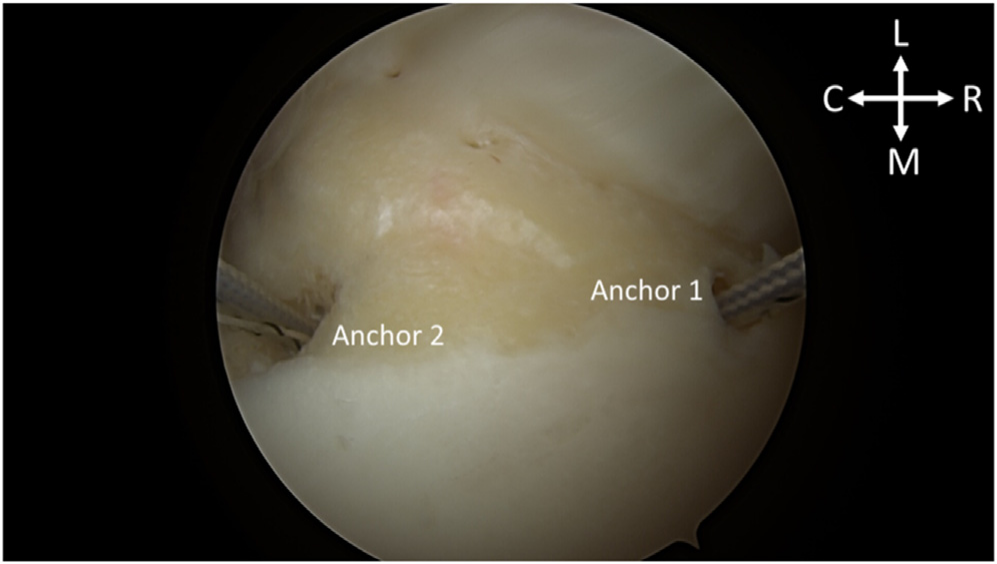
With the patient in the right lateral decubitus position and while viewing from the posterior portal, spinal needle localization, tissue dilation, anchor drilling, and anchor placement are repeated for the more rostral anchor within the Hill-Sachs lesion, noted as Anchor 1 to describe future shuttling. Anchor 1 and the previously placed Anchor 2 can be visualized within the Hill-Sachs lesion adjacent to the humeral head cartilage. (C, caudal; HH, humeral head; HSL, Hill-Sachs lesion; L, lateral; M, medial; R, rostral.)

**Fig 8:**
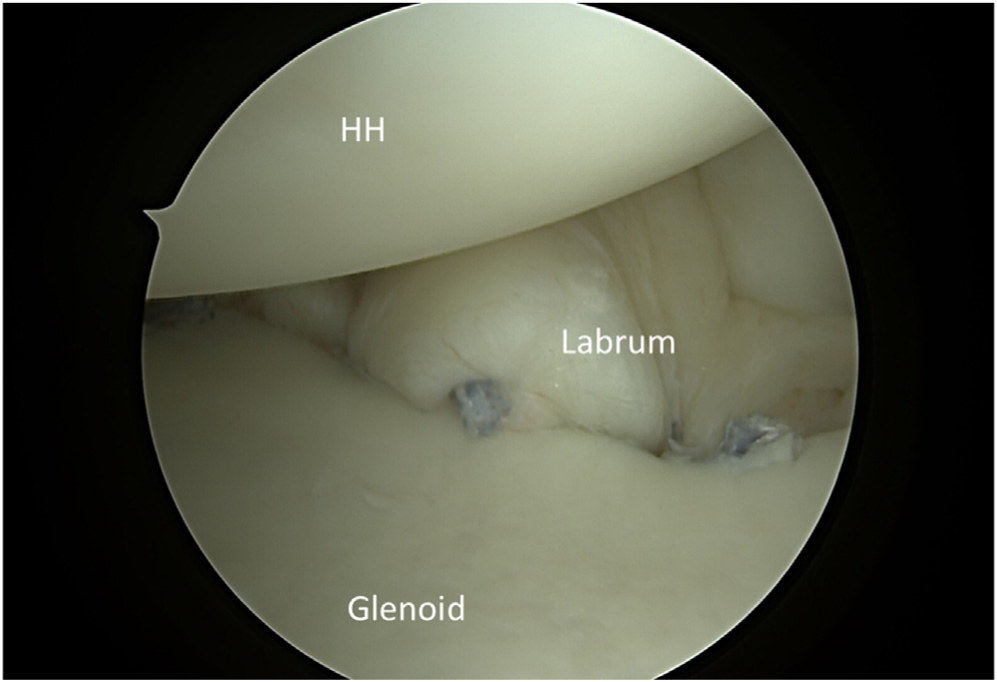
With the patient in the right lateral decubitus position and viewing from the posterior portal, successful completion of the Bankart repair is visualized. (HH, humeral head.)

After portal establishment, the Hill-Sachs lesion is visualized from the posterior portal for remplissage anchor placement (Fig 2). While viewing from the posterior portal, a spinal needle is used to plan the trajectory of the superior and inferior anchor with the arthroscopic portal view (Fig 3). After satisfactory placement of the spinal needle, a small incision is made. Using the Arthrex percutaneous insertion kit for the Knotless 2.6-mm FiberTaks, a nitinol wire is placed through the spinal needle. The needle is removed and a soft tissue dilator is placed over the wire (Fig 4), followed by placement of the drill guide for the anchor being placed over the soft tissue dilator. The nitinol wire and soft tissue dilator are removed, and the drill guide is placed at the area for desired anchor placement (Fig 5). The anchor is drilled and the guide is carefully left in place to facilitate anchor insertion. After successfully malleting in the anchor, the limbs are tensioned, as shown in Figure 6. These steps are then repeated for the superior anchor. These steps are sequentially shown in Video 1. Final anchor placement is shown in Figure 7. After successful placement of the remplissage anchors, attention may then be turned to completing the Bankart repair per surgeon preference. The completed labral repair for this case is presented in Figure 8.

**Fig 9:**
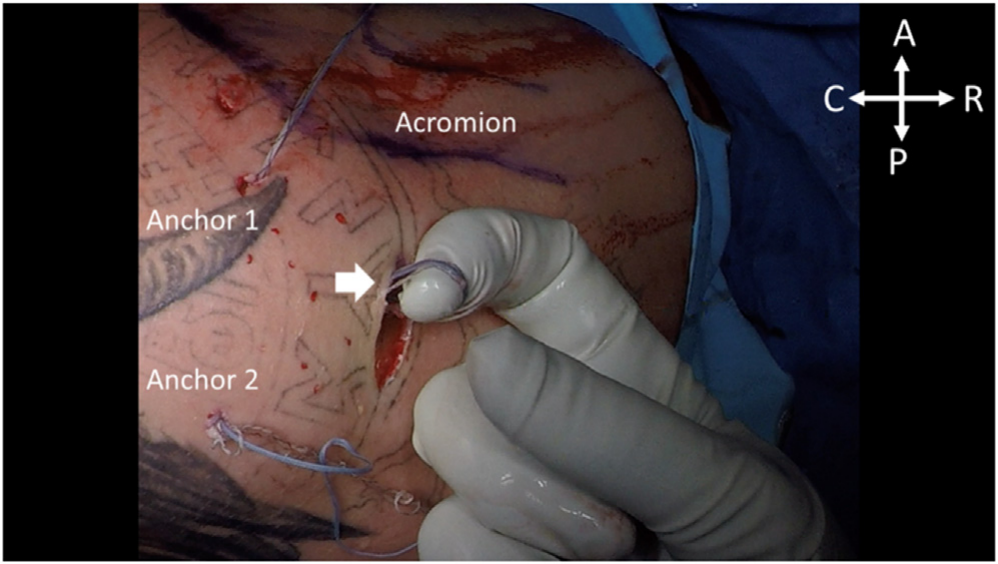
With the patient in the right lateral decubitus position, the posterior viewing portal is expanded and digital dissection is performed to retrieve the sutures from Anchors 1 and 2. The white arrow above indicates the retrieved sutures from Anchor 1 being pulled through the posterior portal. (A, anterior; C, caudal; P, posterior; R, rostral.)

**Fig 10:**
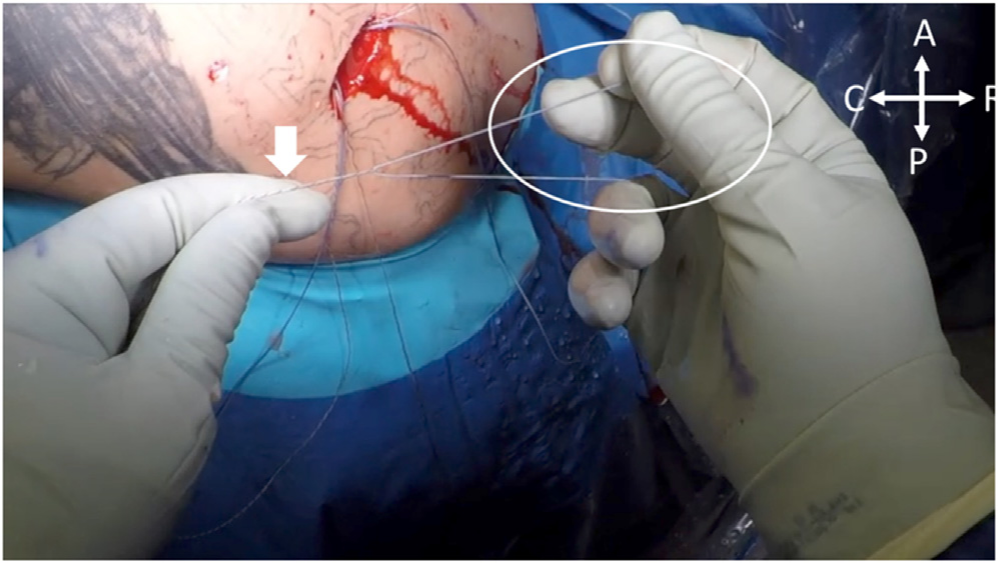
With the patient in the right lateral decubitus position, the double-pulley repair is initiated by taking the repair stitch from Anchor 1, indicated by the white circle, and passing it through the looped shuttle suture of Anchor 2, indicated by the white arrow. (A, anterior; C, caudal; P, posterior; R, rostral.)

**Fig 11:**
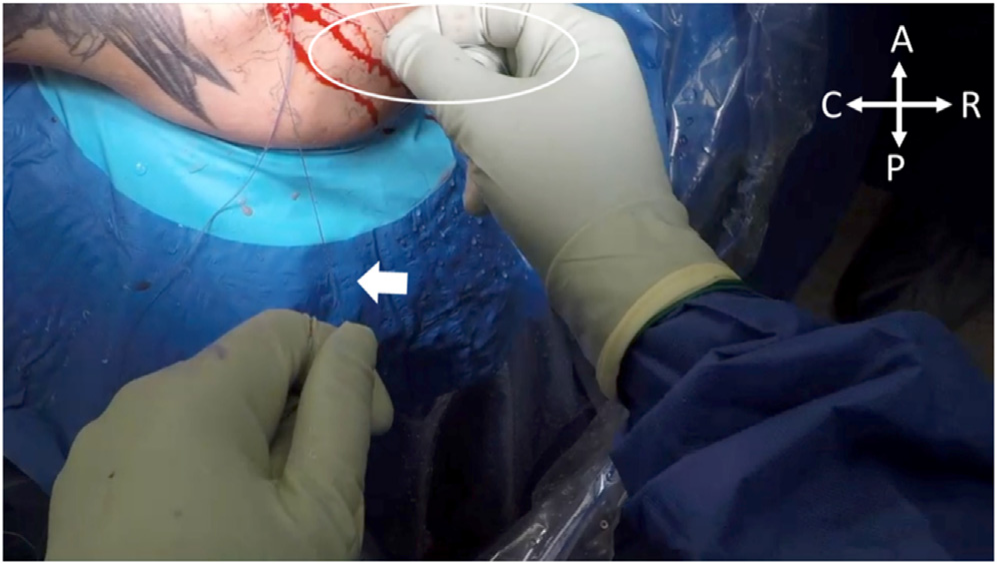
With the patient in the right lateral decubitus position, the nonlooped portion of the shuttle stitch for Anchor 2 (white arrow) is pulled while the repair stitch from Anchor 1, passed through the looped portion of Anchor 2 (circled), is shuttled through the shoulder. (A, anterior; C, caudal; P, posterior; R, rostral.)

**Fig 12:**
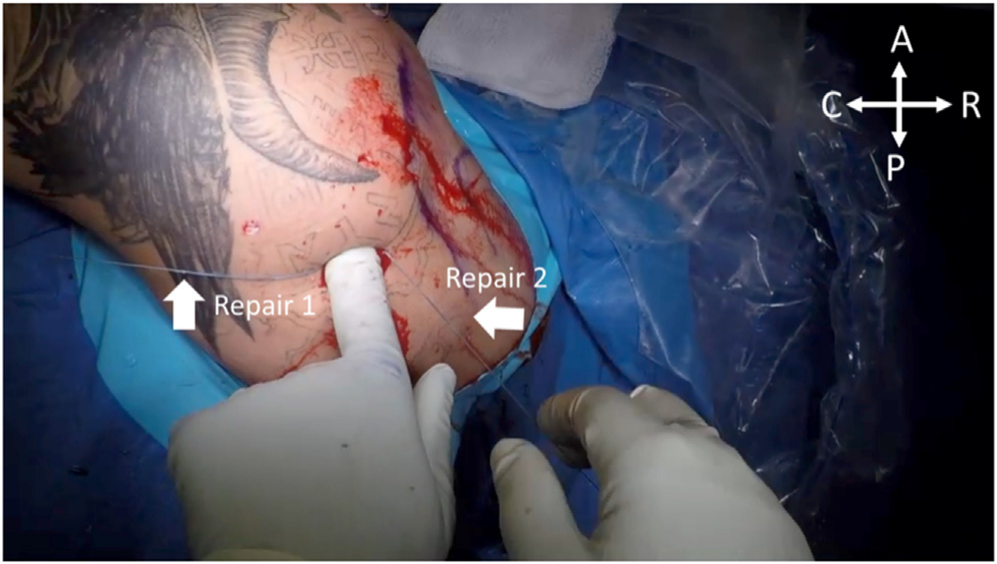
With the patient in the right lateral decubitus position, both repair stitches are successfully shuttled through their accompanying anchor and sequentially tightened to finalize the double-pulley repair. (A, anterior; C, caudal; P, posterior; R, rostral.)

Following completion of the anterior labral repair, attention is then brought back to the Hill-Sachs lesion. The posterior portal is expanded. Digital dissection is performed to the level of the infraspinatus, and the sutures from each anchor are retrieved from the portal and tagged at the superior and inferior aspects of the incision, as shown in Figure 9. Utilizing a double-pulley technique, the repair stitch from each anchor is passed through the looped portion of the shuttle stitch of the opposite anchor. Shown in Figure 10, the repair stitch from the superior anchor, Anchor 1, is passed through the looped portion of the shuttle suture of the inferior anchor, Anchor 2. The nonlooped portion of the shuttle anchor within Anchor 2 is pulled to shuttle the blue repair stitch from Anchor 1 through the shoulder (Fig 11). This process is repeated for shuttling the repair stitch from Anchor 2 through the looped shuttle stitch of Anchor 1. After successful shuttling of both repair sutures, the suture limbs may be sequentially tightened to complete the final repair and then cut via direct palpation, as shown in Figure 12.

## Discussion

This technique presents an alternative method of remplissage that builds upon and refines previous iterations of a double-pulley remplissage.^21^^,^^23^, ^24^, ^25^ Remplissage offers a reliable surgical strategy to decrease recurrence rates of shoulder instability with noncritical bone loss.^5^^,^^10^^,^^14^^,^^15^^,^^26^ When compared to isolated Bankart repair alone, remplissage shows lower recurrence rates, with no significant difference in complication rates or diminishment in range of motion.^15^^,^^26^^,^^27^ This difference remains even when remplissage is compared to open Bankart repairs, as Bitar et al.^28^ found no difference in outcomes between the 2 procedures in a population of contact athletes. Further highlighting the advantage of remplissage, Wu et al.^29^ reported in a biomechanical analysis of bipolar bone loss that remplissage reduced residual anterior shoulder instability to a greater degree than Bankart repair alone. In comparison to bony augmentation procedures, such as a Latarjet procedure, remplissage shows lower recurrence rates of instability and higher return-to-sport rates for appropriately indicated patients.^15^

Given the positive outcomes of remplissage over the past decade, the indications of remplissage have been expanded to patients with on-track lesions at high risk of dislocation.^11^^,^^26^ Thus, the importance of having a reliable and simple technique for performing this procedure is vital. Despite the acknowledgment of the importance of this procedure, a recent international consensus statement has indicated that there is no ideal fixation method for performing a remplissage.^11^ While there is no consensus on ideal fixation, Morrissey et al.^30^ showed that 2 fixation points have higher mean peak resistance to anterior translation compared to 1-anchor constructs, which highlights the importance of the 2-anchor construct presented here. The presented technique offers several advantages to simplify the performance of this procedure, as summarized in Table 1. First, it does not require an accessory posterior portal for anchor placement or suture retrieval. Instead, it allows for percutaneous placement of the anchors to maximize the spread of the anchors, which maximizes filling of the bony defect. Expansion of the previous portal at the conclusion of the procedure simplifies retrieval of the sutures, enhances performance of the double-pulley fixation method, and decreases the likelihood of forming a tissue bridge (Table 2). Furthermore, cutting of the sutures can be done under direct palpation and visualization.

**Table 1:** Advantages and Disadvantages

Advantages	Disadvantages
A wider footprint of fixation may be achieved by percutaneously placing 2 anchors rather than limiting the distance anchors may be placed through a single incision and cannula.	A larger posterior incision is required.
Using the already-made posterior portal prevents the need for a separate portal and facilitates the passage of the sutures for the double-pulley technique.	This procedure cannot be performed in isolation if critical glenoid bone loss is present.
Knotless anchors facilitate the repair by obviating the need for tying knots without sacrificing strength.	
Access to the subacromial space is not required.

**Table 2:** Technical Pearls and Pitfalls

Pearls	Pitfalls
Use of a spinal needle for Hill-Sachs anchor placement while viewing from the posterior portal both maximizes spread and confirms anchors will be retrievable through the posterior incision.	If placing anchors widely apart, both suture limbs must be accessible so they can be digitally retrieved via the expanded posterior portal.
Ensure your finger is deep to the deltoid and superficial to the infraspinatus and retrieve both anchors via the same path to avoid tissue bridge formation.	Ensure a larger hard-bodied anchor is available in the case of anchor pullout or inadequate bone for an all-suture technique.
Place anchors for the remplissage first, before performing labral repair, to improve accessibility and prevent disruption of your labral repair.	When fetching suture, make sure suture limbs remain organized with their respective anchor to avoid shuttling the repair stitch through the same anchor for fixation, creating 2 points of fixation rather than the wide footprint of fixation that this technique is intended to provide.
Tension the remplissage repair following completion of the labral repair instead of before labral repair to avoid loosening of the remplissage.	

The use of a knotless construct is another benefit of this technique. Knotless suture anchor constructs have been shown to have less variability in strength and have been shown to have a higher load to failure than knotted alternatives.^31^^,^^32^ Specifically, for Bankart repair, knotless anchors have been shown to have similar clinical and radiographic outcomes compared to knotted alternatives.^33^ For remplissage, knotted anchors showed a higher incidence of recurrent instability compared to their knotless counterparts.^34^ Therefore, this technique is beneficial in that it decreases the variability of tying knots while not sacrificing strength of fixation or patient outcomes. Without needing to tie knots under direct visualization, this technique also obviates the need to access the subacromial space. The combined benefits of this approach provide a safe, efficient, and effective method of remplissage.

## Disclosures

The authors declare the following financial interests/personal relationships which may be considered as potential competing interests: R.J.F. reports grants and personal fees from Exactech. J.K.E. is a consultant or advisor for Arthrex and Exactech, receives funding grants and travel reimbursement from Arthrex and Exactech, receives nonfinancial support from 10.13039/100007307Arthrex, and is a member of the Editorial Board for *Arthroscopy*. All other authors (C.A., J.A.M., M.H.N., B.L.R.) declare that they have no known competing financial interests or personal relationships that could have appeared to influence the work reported in this paper.

## Supplementary Data

Video 1 This video shows how to perform a simplified double-pulley remplissage with knotless all-suture-anchor fixation using a digital subdeltoid retrieval method. The patient is in the right lateral decubitus position, and anchor placement, through 2 percutaneous portals, is performed while viewing from the posterior portal. After completion of any indicated labral repair, the posterior portal is expanded. The suture limbs are retrieved through this portal via digital dissection, and the repair sutures are passed through the opposite anchor to complete the repair.

## References

[1] Cibulas A., Leyva A., Cibulas G. (2019). Acute shoulder injury. Radiol Clin North Am.

[2] Patrick C.M., Snowden J., Eckhoff M.D. (2023). Epidemiology of shoulder dislocations presenting to United States emergency departments: An updated ten-year study. World J Orthop.

[3] Shields D.W., Jefferies J.G., Brooksbank A.J., Millar N., Jenkins P.J. (2018). Epidemiology of glenohumeral dislocation and subsequent instability in an urban population. J Shoulder Elbow Surg.

[4] Arner J.W., Peebles L.A., Bradley J.P., Provencher M.T. (2020). Anterior shoulder instability management: Indications, techniques, and outcomes. Arthroscopy.

[5] Nazzal E.M., Herman Z.J., Engler I.D., Dalton J.F., Freehill M.T., Lin A. (2023). First-time traumatic anterior shoulder dislocation: Current concepts. J ISAKOS.

[6] Boone J.L., Arciero R.A. (2010). First-time anterior shoulder dislocations: Has the standard changed?. Br J Sports Med.

[7] Buss D.D., Lynch G.P., Meyer C.P., Huber S.M., Freehill M.Q. (2004). Nonoperative management for in-season athletes with anterior shoulder instability. Am J Sports Med.

[8] Dickens J.F., Rue J.-P., Cameron K.L. (2017). Successful return to sport after arthroscopic shoulder stabilization versus nonoperative management in contact athletes with anterior shoulder instability: A prospective multicenter study. Am J Sports Med.

[9] Elser F., Dewing C.B., Millett P.J. (2008). Chondral and osteochondral lesions of the humerus: Diagnosis and management. Oper Tech Sports Med.

[10] Valencia Mora M., Ruiz-Ibán M.Á., Heredia J.D., Ruiz Díaz R., Cuéllar R. (2017). Management of humeral defects in anterior shoulder instability. Open Orthop J.

[11] Hwang S.T., Horinek J.L., Ardebol J., Menendez M.E., Denard P.J. (2022). Arthroscopic remplissage for the treatment of anterior shoulder instability: Current and evolving concepts. JBJS Rev.

[12] Di Giacomo G., Itoi E., Burkhart S.S. (2014). Evolving concept of bipolar bone loss and the Hill-Sachs lesion: From “engaging/non-engaging” lesion to “on-track/off-track” lesion. Arthroscopy.

[13] Mojica E.S., Markus D.H., Colasanti C.A. (2023). Remplissage procedure indications, techniques, and outcomes. Bull Hosp Jt Dis.

[14] Hurley E.T., Matache B.A., Wong I. (2022). Anterior shoulder instability part II—Latarjet, remplissage, and glenoid bone-grafting—an international consensus statement. Arthroscopy.

[15] Davis W.H., DiPasquale J.A., Patel R.K. (2023). Arthroscopic remplissage combined with Bankart repair results in a higher rate of return to sport in athletes compared with Bankart repair alone or the Latarjet procedure: A systematic review and meta-analysis. Am J Sports Med.

[16] Paul R.W., Reddy M.P., Onor G. (2023). Bankart repair with or without concomitant remplissage results in similar shoulder motion and postoperative outcomes in the treatment of shoulder instability. Arthrosc Sports Med Rehabil.

[17] Polio W., Brolin T.J. (2022). Remplissage for anterior shoulder instability: History, indications, and outcomes. Orthop Clin North Am.

[18] Villarreal-Espinosa J.B., Saad Berreta R., Cotter E. (2025). Lower range of recurrent instability rates following Bankart repair and remplissage compared to isolated Bankart repair in patients with “nonengaging/on-track” Hill-Sachs lesions and <20% glenoid bone loss. Arthroscopy.

[19] McNulty A.J., Hartzler R.U. (2023). The triple-double technique of arthroscopic Hill-Sachs remplissage. Arthrosc Tech.

[20] Woodall B.M., Elena N., Paborji D. (2018). Arthroscopic remplissage using a double-pulley system for Hill-Sachs lesions for recurrent shoulder instability. Arthrosc Tech.

[21] McQuivey K.S., Brinkman J.C., Tummala S.V., Shaha J.S., Tokish J.M. (2022). Arthroscopic remplissage using knotless, all-suture anchors. Arthrosc Tech.

[22] Ashy C., Pottanat P., Slone H., Pullen W.M. (2024). Arthroscopic remplissage using a double-pulley technique. Video J Sports Med.

[23] Koo S.S., Burkhart S.S., Ochoa E. (2009). Arthroscopic double-pulley remplissage technique for engaging Hill-Sachs lesions in anterior shoulder instability repairs. Arthroscopy.

[24] Morsy M.G. (2017). Arthroscopic remplissage: Is it still an option?. EFORT Open Rev.

[25] Ratner D.A., Rogers J.P., Tokish J.M. (2018). Use of a knotless suture anchor to perform double-pulley capsulotenodesis of infraspinatus. Arthrosc Tech.

[26] Liu J.N., Gowd A.K., Garcia G.H., Cvetanovich G.L., Cabarcas B.C., Verma N.N. (2018). Recurrence rate of instability after remplissage for treatment of traumatic anterior shoulder instability: A systematic review in treatment of subcritical glenoid bone loss. Arthroscopy.

[27] MacDonald P., McRae S., Old J. (2021). Arthroscopic Bankart repair with and without arthroscopic infraspinatus remplissage in anterior shoulder instability with a Hill-Sachs defect: A randomized controlled trial. J Shoulder Elbow Surg.

[28] Bitar I.J., Allende Nores C., Marangoni L.D., Bustos D.G., Pezzutti L., Bitar L.B. (2025). No difference in outcomes after remplissage or open Bankart repair plus inferior capsular shift in collision and contact athletes with subcritical glenoid bone loss ≤10% and off-track Hill-Sachs lesion. Arthroscopy.

[29] Wu C., Ye Z., Lu S., Fang Z., Xu J., Zhao J. (2024). Biomechanical analysis reveals shoulder instability with bipolar bone loss is best treated with dynamic anterior stabilization for on-track lesions and with remplissage for off-track lesions. Arthroscopy.

[30] Morrissey P.J., Testa E.J., Quinn M. (2025). Both single- and double-anchor remplissage techniques restore native stability in a cadaveric model of Hill-Sachs lesions in anterior shoulder instability. Arthrosc Sports Med Rehabil.

[31] Denard P.J., Adams C.R., Fischer N.C., Piepenbrink M., Wijdicks C.A. (2018). Knotless fixation is stronger and less variable than knotted constructs in securing a suture loop. Orthop J Sports Med.

[32] Hanypsiak B.T., DeLong J.M., Simmons L., Lowe W., Burkhart S. (2014). Knot strength varies widely among expert arthroscopists. Am J Sports Med.

[33] Lobo F.L., Conforto Gracitelli M.E., Malavolta E.A. (2022). No clinical or radiographic difference seen in arthroscopic Bankart repair with knotted versus knotless suture anchors: A randomized controlled trial at short-term follow-up. Arthroscopy.

[34] Michelin R.M., Gornick B.R., Schlechter J.A. (2022). Arthroscopic labral repair of adolescent athlete shoulder instability with knotted versus knotless suture anchors. Orthop J Sports Med.

